# Hamstring Myometric Properties and the Functional Outcome in Young Adults with Radicular Pain: A Cross-Sectional Study

**DOI:** 10.5114/jhk/196356

**Published:** 2025-05-29

**Authors:** Tomasz Kuligowski, Błażej Cieślik

**Affiliations:** 1 Faculty of Physiotherapy, Wroclaw University of Health and Sport Sciences, Wroclaw, Poland.; 2 Healthcare Innovation Technology Lab, IRCCS San Camillo, Venice, Italy.

**Keywords:** pain, biomechanics, sciatica, myometric analysis

## Abstract

Low back pain (LBP) is a widespread musculoskeletal condition caused by various pathologies, including peripheral nervous system dysfunction, resulting in stiffness, reduced strength, and compromised neuromuscular control. Therefore, this study aimed to evaluate the influence of single-leg radicular pain on hamstring biomechanics in young adults without significant concurrent lower back symptoms while investigating the association between the functional outcome and muscle biomechanical properties. This cross-sectional study included two groups: young adults with lower limb radicular pain (RAD, n = 15) and age-matched healthy asymptomatic individuals (CON, n = 15). Clinical characteristics were assessed using the Oswestry disability index (ODI), the numeric pain rating scale (NPRS), and the passive straight leg raise (PSLR) test. Myometric muscle analysis of the biceps femoris and semitendinosus muscles was conducted using the Myoton PRO® device, focusing on mechanical and viscoelastic properties such as frequency, stiffness, relaxation time, decrement, and creepability. The RAD group showed around 14% higher stiffness on the symptomatic side compared to the CON group (p = 0.003), while relaxation time and creepability were by approximately 14% (p = 0.008) and 13% (p = 0.01) lower, respectively. Similarly, the RAD group exhibited approximately 13% higher stiffness on the asymptomatic side compared to the CON group (p = 0.02). In conclusion, the biomechanical properties of the hamstrings—particularly stiffness, relaxation time, and creepability of the biceps femoris on the symptomatic side—may significantly impact pain management in individuals with radicular pain. Moreover, stiffness of the biceps femoris could be an important predictor of functional outcomes in younger adults.

## Introduction

Low back pain (LBP) is one of the most common musculoskeletal diseases that affect the population of the world (Chou, 2021; Knezevic et al., 2021). It can be caused by many pathologies, such as the overload or trauma (Chou et al., 2007). Those pathologies can lead to many miscellaneous disorders and disability in daily activities and manifest in one or more, often overlapped, types of pain: nociceptive, nociplastic, neurogenic or nonspecific, locally or peripherally to its source (Nijs et al., 2015). Whether local or referred, somatic pain is elicited through noxious stimulation of local nerve endings. Neuropathic pain, a significant mimic, arises from ectopic impulses originating in the axons of a peripheral nerve. On the other hand, central pain is engendered by the activity of second-order or third-order neurons within the nociceptive system. Referred pain can manifest in a region distant from the main lesion and may occasionally be linked to secondary hyperalgesia and trophic alterations in the areas where the pain is referred. Radicular pain is precisely characterised by its mechanistic origin, indicating pain elicited through stimulation of a spinal nerve's sensory (dorsal) root or its associated dorsal root ganglion. Despite notable ambiguity, alternative definitions based on clinical features are prevalent in the medical literature. The clinical delineation of radicular pain is indistinct, marked by a diverse array of subjective descriptions of the pain's qualitative aspects. Furthermore, the anatomical distribution of radicular pain exhibits considerable variability owing to divergent levels of affected nerve roots and the inherent normative diversity in dermatomal cutaneous supply among individual patients (Dower et al., 2019). Lumbar discopathy (LD) is the leading cause of diagnosed LBP (Zhang et al., 2023). LD plays a crucial role in nerve root compression syndromes that can result in sciatica and other types of neurodynamic disorders (Park et al., 2020; Pesonen et al., 2021a). Therefore, a person with such a pathology may experience constant or intermittent pain, sensory, reflexes, and muscle strength deficits during or without movement, which, among others, also can increase the risk of peripheral joint injuries (Vermeulen et al., 2023). Other causes of radicular pain include vertebral degenerative, other vascular, cystic, infectious, neuromeningeal and neoplastic causes (Bogduk and Govind, 1999).

Presently, our comprehension suggests that the peripheral nervous system (PNS) malfunction may affect muscle tissue, resulting in increased stiffness, diminished strength, and compromised neuromuscular control (Chou, 2021). This impact is particularly significant in the hamstrings and hip adductor muscles due to their direct innervation from the sciatic nerve. This impairment can be observed as changes in muscle tissue biomechanical (stiffness, decrement) and viscoelastic (relaxation, creep) properties (Alcaraz-Clariana et al., 2021). Each nerve root's sliding ability has its limit. Therefore, any disc pathology that causes foraminal stenosis results in nerve root impingement, impairing its ability to move along other tissues (López-de-Celis et al., 2022; Nee et al., 2022). This can cause increased mechanosensitivity, which limits the posterior thigh range of motion (ROM). Moreover, sacral plexus neurodynamic disorders can affect lower body functioning (Bonser et al., 2017; Pesonen et al., 2021b). For this reason, neurodynamic treatment has been found to be more efficient in improving the ROM of the hamstring muscles compared to the standard sustained stretch (Bonser et al., 2017; de Ridder et al., 2020; Satkunskiene et al., 2020).

Expanding on the relationship between muscle mechanical disorders and peripheral nerve diseases, it is crucial to realize that peripheral nerve dysfunction can directly influence muscle properties due to altered neurodynamics (Bogduk, 2009). Physiological mechanisms underlying this relationship include impaired nerve gliding, increased mechanosensitivity, and altered neural signaling, which can lead to muscle stiffness, decreased elasticity, and changes in muscle tone (Butler and Coppieters, 2007). When a nerve is compressed or irritated, its ability to transmit signals efficiently is compromised, leading to abnormal muscle contractions, reduced flexibility, and even muscle atrophy over time. These alterations in muscle mechanics are significant because they can exacerbate pain, limit range of motion, and impair overall function (Graven-Nielsen, 2006). For instance, in radicular pain, impaired nerve dynamics can result in increased muscle stiffness, further restricting movement and potentially leading to compensatory injuries in other parts of the body. Understanding these mechanisms is crucial for developing comprehensive treatment strategies. Addressing both the neural and muscular components of the disorder could lead to more effective interventions, reducing pain and improving function (Lee et al., 2024). This integrated approach is essential for optimizing patient outcomes, particularly in conditions where the nerve dysfunction plays a central role in musculoskeletal disorders (Hori et al., 2021; Patel and Perloff, 2018; Raftry and Marshall, 2012).

Many clinical tests have been established to differentiate, diagnose, and evaluate the source of the pain, the level of spine potential damage, and the severity of the pathology. Slump and straight leg raise (SLR) tests are most widely used in clinical practice when patients report peripheral pain and/or neurological deficits from the sacral plexus or dura mater (Capra et al., 2011; Majlesi et al., 2008). Nevertheless, several tests should always be used to obtain the complete clinical outcome of the patient, including the anamnesis (Petersen et al., 2017). The subjective diagnosis may be supplemented by myometric analysis, a quantitative assessment technique that involves measuring and analyzing the contractile properties of muscles (Lough et al., 2000). By examining factors such as force generation, contraction velocity, and muscle relaxation, myometric analysis provides valuable insights into muscle performance and the identification of potential abnormalities (Mencel et al., 2021).

Neurodynamics is a clinical concept that involves utilizing movement in two ways: firstly, to evaluate heightened mechanosensitivity of the nervous system, and secondly, to restore the disrupted homeostasis within and surrounding the nervous system. Proper neurodynamics of the peripheral nervous system requires the nerve to have spatial ability of three-dimensional, unimpaired gliding/sliding between adjacent tissues (De-la-Llave-Rincon et al., 2012; Sierra-Silvestre et al., 2018). The ability of the nervous system to adapt to mechanical loads is crucial. It undergoes various mechanical events such as elongation, sliding, cross-sectional change, angulation, and compression. If these dynamic protective mechanisms fail, the nervous system becomes susceptible to neural edema, ischemia, fibrosis, and hypoxia, leading to altered neurodynamics (Ellis and Hing, 2008). Mobilizing a nerve can potentially decrease the internal pressure within the nerve, leading to enhanced blood circulation. This process can promote axonal transport and nerve conduction. Likewise, elongating the nerve bed stimulates nerve gliding, which raises nerve tension and intraneural pressure (Alshami et al., 2021; Coppieters and Butler, 2008).

To our knowledge, limited research has focused on the biomechanics and neurodynamics of lower extremity muscles, including the hamstrings, in individuals with low back pain (Bueno-Gracia et al., 2019; Halbertsma et al., 2001). Additionally, there is a lack of scientific data on the mechanical properties of knee flexors in young adults affected explicitly by unilateral radicular pain without additional dominant symptoms in the lower back (Miyamoto et al., 2018; Miyamoto and Hirata, 2019). Therefore, this study primarily aimed to evaluate the influence of single-leg radicular pain on hamstring biomechanics in young adults without significant concurrent symptoms in the lower back. Additionally, the study aimed to investigate the association between the participants' functional outcome and the biomechanical characteristics of their muscles.

## Methods

### Study Design

This study was designed as a cross-sectional study, prepared in accordance with the STROBE guidelines, and conducted between January and May 2022 (Cuschieri, 2019). The study protocol received prior approval from the Institutional Review Board of the Wroclaw University of Health and Sport Sciences, Poland (protocol code: 7/2022; approval date: 27 March 2022). Prior to the commencement of the research, all subjects were provided with information regarding the study procedures and their right to decline or withdraw at any time. Informed consent was obtained from all participants prior to their study enrollment.

### Participants

The radicular pain (RAD) group consisted of patients who sought treatment for lower limb radicular pain at a private physiotherapy clinic, while the control (CON) group comprised age-matched healthy asymptomatic individuals recruited from the university. The inclusion criteria were individuals aged between 18 and 35 years, who reported unilateral gluteal or posterior thigh radicular pain with a minimum intensity of 3/10 on the numeric pain rating scale (NPRS), resulting in subjective disability in activities of daily living, that lasted for at least six weeks. Exclusion criteria consisted of severe spinal pathology (e.g., metastatic disease, fracture), severe spinal deformity, history of spinal, hip or knee area surgery, a lower limb muscle pathology (e.g., trauma), neoplasm or other disorders that could influence the outcomes, as well as participation in professional-level sports activities or being under any treatment.

Finally, the study comprised 30 participants, including 21 females (70%) and 9 males (30%). The mean age of female participants was 28.67 years (SD: 4.83) with a BMI of 22.14 (SD: 2.19), while the mean age of male participants was 25.22 years (SD: 3.87) with a BMI of 21.92 (SD: 1.62). Pain intensity, measured by the NPRS, averaged 2.86 (SD: 2.74) for females and 2.78 (SD: 2.54) for males, while the Oswestry Disability Index (ODI) scores were 27.14% (SD: 17.70) and 27.56% (SD: 20.09), respectively.

### Outcome Measures and Procedures

A licensed physiotherapist with over 10 years of experience in manual therapy, adhering to the International Federation of Orthopaedic Manipulative Physical Therapists (IFOMPT) standards, conducted all assessments throughout the study.

### Myometric Analysis

All participants underwent evaluation of the hamstrings (semitendinosus, SEMI, and biceps femoris, BF) using the noninvasive Myoton PRO® device (Myoton AS). It has been widely used and is considered a safe, reliable, and noninvasive device for assessing muscle biomechanics and viscoelastic properties (Aird et al., 2012; Lettner et al., 2024; Li et al., 2022). Several variables were measured bilaterally, including the state of tension (natural oscillation frequency), biomechanical properties (dynamic stiffness, logarithmic decrement of natural oscillation, characterizing elasticity), and viscoelastic properties (mechanical stress relaxation time, and a ratio of deformation and relaxation time, characterizing creep). The frequency (Hz) reflected muscle tone or tension; a higher value denoted increased tone possibly from conditions such as spasticity or muscle shortening. Dynamic stiffness (N/m) assessed muscle pliability; a higher value signified stiffness potentially due to fibrosis or scarring. Logarithmic decrement measured muscle elasticity; a higher value suggested reduced elasticity due to factors like aging or scarring. Mechanical stress relaxation time (ms) gauged muscle response post external force; a higher value meant a longer relaxation time, often suggesting reduced compliance or increased viscosity. Lastly, creepability evaluated the viscoelastic property; a higher value implied a more viscous tissue potentially from inflammation or fibrosis.

**Figure 1:**
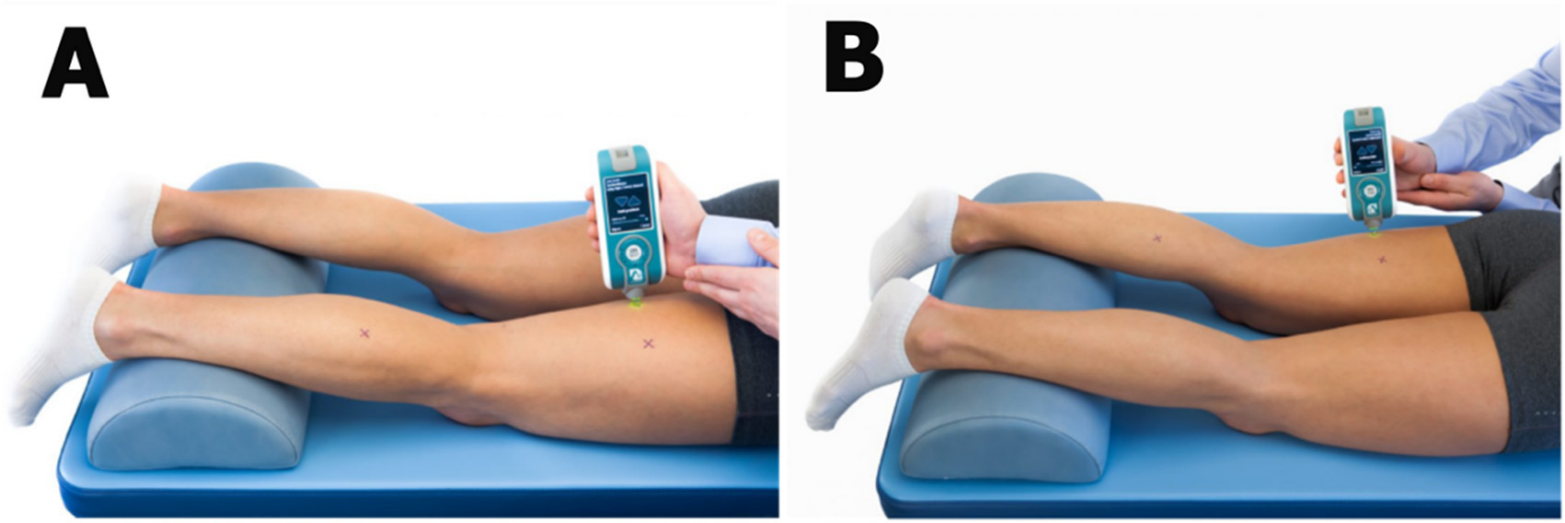
Myoton device appliance within semitendinosus muscle (A) and biceps femoris muscle (B). Source: www.myoton.com (accessed on 07 August 2023)

The participant was positioned prone, with an oval pillow placed underneath their ankle joints to relax their hamstrings, and was instructed not to move until the procedure was concluded. All procedures followed the manufacturer’s guidelines (Figure 1), following a 5-min period during which participants remained still. The measurements, conducted by the same researcher, were performed three times on each side within the SEMI and the BF, and a mean score was utilized. Participants were instructed to refrain from engaging in stretching or warm-up activities for at least 24 h prior to the measurements. To ensure accurate myotonometric measurements and prevent technical artifacts, the manufacturer of the Myoton device has integrated real-time guidance features that detect unwanted motion, muscle contractions, excessive compression force, and other potential issues. Additionally, our evaluator maintained a stable arm position, resting their elbow on a leveled table to mitigate any undesirable effects. The room temperature was constant at 22°C.

### Functional and Neurological Assessment

The PSLR test was used to assess the neurodynamics of the sciatic nerve. The participant was positioned supine. This test was performed based on the literature and scored positive when participants’ symptoms in the thigh within hip flexion less than 60 degrees were provoked. Above those values, it was scored as negative (Boyd et al., 2013; Capra et al., 2011). Each time, the differential diagnosis using additional distal joint maneuvers to exclude false-positive results due to possible muscular disorder was used. The degree value was measured with a goniometer, and the examination table was levelled before the procedure. Moreover, each participant was neurologically assessed to the extent available, as this is part of the evaluation and differentiation for radicular pain and radiculopathy. The examination included the assessment of the Achilles tendon reflex (for evaluating the S1 root), the strength of the extensor hallucis longus muscle (for the L5 root), and the evaluation of superficial sensation along the L5 and S1 dermatome.

### Oswestry Disability Index (ODI) Questionnaire

The ODI questionnaire was used to assess the level of subjective disability, including its impact on activities of daily living (ADLs). It has ten sections for daily activities, scoring between 0 and 5 points each. The overall score varies from minimal disability (0 to 20%) to bedboundness (80 to 100%). The questionnaire was used in Polish (Fairbank and Pynsent, 2000).

### Pain Intensity Measurement

The Numeric Pain Rating Scale (NPRS), an 11-point scale, was used to measure pain intensity in participants from the RAD group on the same day as the ODI. The NPRS was applied according to the procedure described previously (Chiarotto et al., 2018, 2019).

### Data Analysis

Data were analyzed using JASP version 0.16.3 (University of Amsterdam, The Netherlands). Categorical variables were described as frequency counts and percentages, while continuous variables were reported as means and standard deviations (SDs). The Shapiro-Wilk test was used to assess the distribution of quantitative data. For continuous variables, unpaired *t*-tests (when normal distribution) or *U* Mann-Whitney tests (when non-normal distribution), along with *χ*^2^ tests for categorical data, were employed to compare baseline demographic variables between groups. To analyze the differences in myometric variables between groups, an unpaired *t*-test was used. Multiple linear regression (stepwise) was employed to identify the association between clinical test results and biomechanical muscle variables. Prior to conducting the multiple linear regression analysis, the assumption of a linear relationship (using pairwise Pearson *r* test) between the outcome variable and the independent variables was assessed. Statistical significance was set at *α* < 0.05.

## Results

### Participants Characteristics

**Figure 2:**
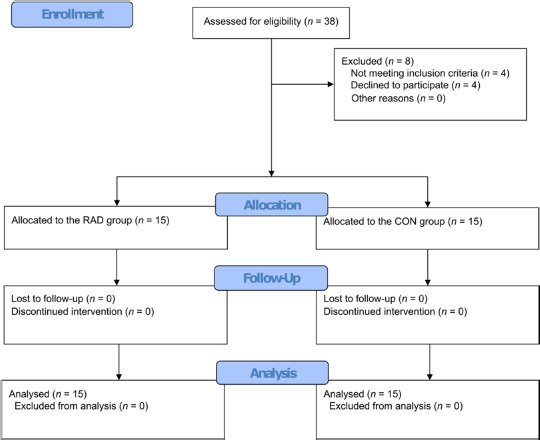
Study flow diagram.

Out of the 38 potential participants, 30 met the inclusion criteria and were included in the study. All enrolled participants successfully completed the study and were included in the analysis (Figure 2). Table 1 shows no statistically significant differences between the RAD and CON groups within age and anthropometric variables. However, there were significant differences between the groups regarding functional outcomes. The RAD group had an ODI of 44.27 (SD 5.44) and a mean pain level of 5.27 (SD 1.10), while the CON group had an ODI of 10.27 (SD 5.34) and a mean pain level of 0.40 (SD 0.74).

**Table 1:** Participants’ characteristics.

Variable	Total	RAD	CON	*p* value
*n*	30	15	15	-
*n* (%) females	21 (70.00)	11 (73.33)	10 (66.66)	0.69^a^
Age, years	27.63 (4.77)	28.53 (5.62)	26.73 (3.73)	0.31^b^
Body mass, kg	63.63 (7.53)	63.20 (7.51)	64.07 (7.79)	0.76^b^
Body height, m	1.70 (0.07)	1.69 (0.06)	1.70 (0.08)	0.61^b^
Body mass index, kg/cm^2^	22.07 (2.01)	22.04 (2.10)	22.11 (1.98)	0.93^b^
ODI, %	27.27 (18.10)	44.27 (5.44)	10.27 (5.34)	< 0.001^c^
NPRS, 0–10	2.83 (2.64)	5.27 (1.10)	0.40 (0.74)	< 0.001^c^
PSLR symptomatic, ^o^	48.83 (14.40)	38.47 (7.97)	59.20 (11.64)	< 0.001^c^
PSLR asymptomatic, ^o^	47.57 (13.71)	37.73 (6.31)	57.40 (11.93)	< 0.001^c^

ODI: Oswestry disability index; NPRS: numeric pain rating scale; PSLR: passive straight leg raise; ^a^ Chi-square test; ^b^ t-test; ^c^ Mann-Whitney test; values presented as mean (SD)

### Between-Group Differences in Muscle Biomechanics

Significant differences were observed between the groups in terms of myometric analysis results for both the symptomatic and asymptomatic sides (Table 2). On the symptomatic side, the RAD group exhibited approximately 14% higher stiffness compared to the CON group (241.67 vs. 207.07; *p* = 0.003), while relaxation time and creepability were lower by approximately 14% (22.88 vs. 26.11; *p* = 0.008) and 13% (1.39 vs. 1.57; *p* = 0.01), respectively. On the asymptomatic side, the RAD group showed approximately 13% higher stiffness compared to the CON group (239.93 vs. 209.73; *p* = 0.02). However, there were no significant differences between the groups in other myometric variables.

**Table 2:** Myometric variables of between-group differences in relation to the symptomatic and the asymptomatic side.

Variable			RAD (*n* = 15)	CON (*n* = 15)	*p* value
**Symptomatic side**					
	Biceps Femoris				
		Frequency, Hz	13.69 (1.40)	12.85 (1.32)	0.10
		Stiffness, N/m	241.67 (34.94)	207.07 (21.05)	0.003
		Decrement, log	1.23 (0.39)	1.14 (0.15)	0.41
		Relaxation time, ms	22.88 (3.44)	26.11 (2.65)	0.008
		Creepability, De	1.39 (0.21)	1.57 (0.15)	0.01
	Semitendinosus				
		Frequency, Hz	13.57 (1.69)	13.31 (1.57)	0.67
		Stiffness, N/m	231.40 (39.32)	211.67 (34.16)	0.15
		Decrement, log	1.22 (0.39)	1.18 (0.18)	0.71
		Relaxation time, ms	23.18 (4.02)	24.60 (3.52)	0.31
		Creepability, De	1.41 (0.27)	1.56 (0.15)	0.07
**Asymptomatic side**					
	Biceps Femoris				
		Frequency, Hz	13.44 (1.59)	12.80 (1.20)	0.22
		Stiffness, N/m	239.93 (41.91)	209.73 (19.02)	0.02
		Decrement, log	1.22 (0.39)	1.14 (0.16)	0.47
		Relaxation time, ms	23.34 (4.34)	25.96 (2.50)	0.05
		Creepability, De	1.41 (0.27)	1.56 (0.15)	0.07
	Semitendinosus				
		Frequency, Hz	13.54 (1.32)	13.37 (1.43)	0.74
		Stiffness, N/m	231.93 (33.84)	210.87 (27.83)	0.07
		Decrement, log	1.21 (0.35)	1.19 (0.19)	0.82
		Relaxation time, ms	22.76 (3.28)	24.71 (2.72)	0.09
		Creepability, De	1.36 (0.20)	1.46 (0.14)	0.13

Values presented as mean (SD); p value as a result of the paired t-test

### Correlation and Predictors Results

**Figure 3:**
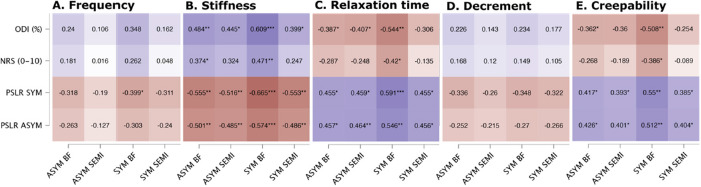
Correlation results for (A) frequency, (B) stiffness, (C) relaxation time, (D) decrement, and (E) creepability. * p < 0.05, ** p < 0.01, *** p < 0.001

Figure 3 illustrates the correlation heatmap between the functional outcome and the myometric muscle variables. Significant correlations were observed for both the symptomatic and asymptomatic sides, particularly in hamstrings stiffness (ranging from −0.66 to 0.37), relaxation time (ranging from −0.59 to 0.42), and creepability (ranging from −0.55 to 0.36). Regarding stiffness, higher values were associated with higher scores on the ODI and NPRS, as well as lower scores on the PSLR test. Conversely, lower values in relaxation time or creepability corresponded to higher ODI and NPRS scores and lower PSLR test scores.

To evaluate the extent to which myometric analysis can explain the statistically significant variance in participants' functional outcomes, a stepwise multiple regression analysis was performed. Predictors included in the regression models were stiffness, frequency, relaxation time, decrement, and creepability of both the symptomatic and asymptomatic extremities, including the biceps femoris and semitendinosus muscles. In terms of the ODI and NPRS results, it was observed that stiffness of the symptomatic side biceps femoris served as an important predictor variable, accounting for 37% and 22% of the variance in these models, respectively (*p* < 0.001 and *p* = 0.009, respectively) (Table 3). The *B* value indicates that for every one-unit increase in the symptomatic biceps femoris stiffness (independent variable), there was a corresponding increase of 0.33 units in the ODI score and 0.04 units in the NPRS score (dependent variables).

**Table 3:** Stepwise regression results.

Variable		*B*	Beta	*t*	*p* value	*F*	R^2^
**ODI**					< 0.001	16.54	0.37
	Symptomatic side biceps femoris stiffness	0.33	0.61	4.07			
**NPRS**					0.009	8.00	0.22
	Symptomatic side biceps femoris stiffness	0.04	0.47	2.83			

ODI: Oswestry disability index; NPRS: numeric pain rating scale

## Discussion

This study aimed to assess how single-leg radicular pain affected the biomechanical and viscoelastic properties of the hamstring muscles in young adults, particularly in the absence of significant lower back symptoms. The results indicated that radicular pain led to measurable changes in muscle stiffness, relaxation time, and creepability, especially in the biceps femoris, even when lower back pain was not present.

Lower back disorders may vary in terms of symptoms. Those can be related to the lumbopelvic area or extremities or both. Most LD can cause neurodynamic malfunction within the dura mater or nerve roots which in some instances may be asymptomatic to the individual experiencing it in ADLs. The occurrence of symptoms depends on multifactorial background; however, due to better hydration of the discs the likelihood of asymptomatic condition is higher in younger people (Ashinsky et al., 2021; Yang et al., 2020; Żak and Pezowicz, 2016). Currently there is a consensus on the theoretical effects of different spinal pathologies on individual functional outcomes. However, it remains unclear whether the myometric properties of the hamstring vary in young people with radicular pain (RP) but without experiencing lower back aches. Of note, a radicular pain term should be understood and separated from radiculopathy as the first refers to gluteal or extremity pain usually with no neurological deficits observed (Bogduk, 2009; Oliveira et al., 2020).

This study showed a number of patterns that can confirm impairment of the PNS within its neurodynamics in people with single-leg RP. In line with previous studies, it was confirmed that this sort of disorder significantly influenced the individuals’ functional outcome (Halicka et al., 2022; Nee et al., 2022). Firstly, the PSLR test result significantly differed in people with RP. This mechanism has been previously described in the literature widely (Murape et al., 2022; Peacock et al., 2023; Pesonen et al., 2021b; Tawa et al., 2017). It relates to nerve structures gliding impairment which can be observed in discopathy or other pathology causing foraminal impingement (Coppieters and Butler, 2008; Neto et al., 2018). While healthy individuals were close to normative values of the PSLR test (60^o^ and above), the symptomatic ones mainly were below 40°, which clinically can be interpreted as having significant neurodynamic disorder. Similar observations were also made by other investigators (Majlesi et al., 2008; M’kumbuzi et al., 2012; Rade et al., 2017).

Nevertheless, the PSLR values were also below normative on the asymptomatic side within the RP individuals, which can suggest that pathology of a single sciatic nerve can affect the other. Secondly, the subjective disability outcome of individuals who experienced RP has also been considered severer. Neurodynamic dysfunction can be problematic predominantly within walking as hip flexion with the extended knee is limited with pain. Although previous research has confirmed that lower back disorders significantly affect the gait and lumbopelvic function, the fact of limited data on radicular pain effects on the gait must also be highlighted (Natarajan et al., 2022; Smith et al., 2022; Tsigkanos et al., 2021). Thirdly, pain during ADLs has been apprised to be more assertive in individuals with RP, which is also in line with previous research (Lee et al., 2019).

The critical point of this study, however, is that the myometric properties of hamstrings in individuals with RP provided some new insights. Firstly, to provide the overall look, it should be highlighted that every single variable in the symptomatic group had its pattern in both BF and ST muscles. Nonetheless, only the BF within the symptomatic group showed some statistical significance. There is a lack of data in the literature to compare this observation and explain clearly the differences between the BF and the ST; however, it can be related to biomechanics and anatomy, as other authors recommend considering hamstrings as three different muscles where one can compensate for the others in some aspects, despite the fact of the same innervation (Farfán et al., 2021; Kawama et al., 2022; Lempainen et al., 2021). Additionally, it was suggested that among hamstrings, the BF could be mainly activated during an eccentric contraction, such as walking (Guruhan et al., 2021; Llurda-Almuzara et al., 2021). Frequency, elasticity, and stiffness values were usually higher than in the control group, while creep and relaxation were lower. This confirms the physiological phenomenon where the higher decrement means the lower elasticity of the tissue.

Increased stiffness is likely due to impaired neurodynamics, where nerve root compression or irritation reduces the nerve's ability to glide, resulting in increased muscle tension. Interestingly, the asymptomatic side in the RAD group also exhibited higher stiffness compared to the CON group, suggesting that unilateral radicular pain might have a bilateral impact on muscle properties. In terms of elasticity, the study observed a decrease in the symptomatic leg's elasticity, as reflected by a higher logarithmic decrement. Reduced elasticity can lead to a decreased ability of the muscle to return to its original shape after being stretched, which could further contribute to functional limitations in individuals with radicular pain. This finding emphasizes the importance of addressing elasticity issues in rehabilitation programs to restore normal muscle function. Muscle tone, indicated by natural oscillation frequency, was found to be higher in the symptomatic leg. This suggests that muscles in the affected limb are in a state of increased tension, possibly due to prolonged nerve irritation. Elevated muscle tone can lead to muscle fatigue and discomfort, exacerbating the individual’s overall symptoms and functional limitations (Kamandulis et al., 2024). This reinforces the need for targeted interventions aimed at reducing muscle tone to alleviate pain and improve mobility. Finally, the study found that both relaxation time and creepability were reduced in the symptomatic leg, indicating altered viscoelastic properties. Shorter relaxation time and decreased creepability reflect the muscle's impaired ability to relax and deform, respectively, under sustained loads. These changes can contribute to increased pain and reduced range of motion, further impacting the patient's quality of life. The bilateral presence of these changes, although more pronounced on the symptomatic side, suggests that radicular pain may have more widespread effects than previously thought.

It should be also noted that the asymptomatic leg had the same pattern as the symptomatic one. Again, frequency, elasticity, and stiffness values were higher, while creep and relaxation were lower in RP individuals compared to healthy ones; however, no statistical significance was observed within any of these variables. This provides a new perspective on single-leg radicular pain. A larger sample size is necessary to explain this observation; however, this suggests that the source of the pathology (discopathy, bone spurs, tumor, fracture or other foraminal compromise background) may impair the PNS bilaterally without symmetric symptoms occurrence.

Taken together, this study showed that neurodynamic disorder might affect young people not only by worsening their functional outcome, but also by disturbing the physiological mechanisms of muscle tissue.

### Limitations and Future Study Directions

This study showed perspectives, directions, and tendencies of the PNS neurodynamics in individuals with RP. However, given the small sample size and the lack of a priori sample size calculation, caution must be applied when interpreting the results. A significant limitation of this cross-sectional study is the lack of control for potential confounding factors. While we aimed to explore associations between clinical and biomechanical variables, we did not account for other variables that could influence these relationships, such as participants' physical activity levels, comorbid conditions or medication use. Additionally, the present study focused solely on young adults, with the majority of included participants being women, and we did not collect data on specific phases of the menstrual cycle or pregnancy status. The important limitation is that no lumbar MRI and any other imaging had been performed prior to the enrollment; thus, the precise source of the pathology had not been determined, which could potentially suggest a different point of view. Moreover, while we applied stringent inclusion criteria and differential diagnosis using the PSLR test to identify radicular pain, we did not gather a comprehensive self-reported pain history or utilize specialized pain assessment tools beyond the NPRS. Furthermore, a disadvantage of the study is that the results indicate an immediate effect, which may reflect a thixotropic impact on the measured tissue response. The magnitude of this thixotropic effect, as described by Simons and Mense (1998), remains unclear and warrants further investigation. Another limitation of this study is that we did not measure lumbar, hip, and knee range of motion to exclude joint restrictions, potentially impacting the comprehensiveness of our findings. Also, further research is needed to explore how the dominant limb may contribute to radicular pain and whether it influences the frequency of pain occurrence.

For future research, it is essential to broaden the age range for enhanced generalizability. Implementing lumbar MRI and other diagnostics would pinpoint the pathology's origin more accurately. Deepening our inquiry by collecting detailed pain histories and using specialized assessment tools would refine the quality of our findings. It is also pivotal to increase the sample size for robust conclusions, and exploring other pertinent variables would provide a holistic understanding of PNS neurodynamics in RP individuals. Finally, future studies should incorporate strategies to identify and control for confounding factors to enhance the accuracy and reliability of the results.

## Conclusions

The results of this study reveal that in young individuals with single-leg radicular pain, the biomechanical and viscoelastic properties of the hamstring undergo changes due to neurodynamic disorders, even when no local lower back pain is evident. Notably, stiffness and relaxation time are pivotal factors in this context. This knowledge significantly enriches our understanding of this pathology. It suggests that when strategizing treatments, we must adopt a broader perspective since myometric changes are evident on both sides. Moreover, stiffness in the biceps femoris of the symptomatic leg could be a critical indicator of functional outcomes in younger adults. These insights have profound clinical implications, guiding practitioners in their treatment approach. Consequently, patients should be viewed as having a bilateral disorder, even if symptoms only manifest on one side during initial stages. Following this, interventions that incorporate two-sided or central neural techniques might be more effective. While our findings are promising, they would benefit from validation through a study with a more extensive sample size to extend our knowledge of this condition.
